# Perioperative blood transfusion and the prognosis of ovarian cancer surgery: A meta-analysis

**DOI:** 10.12669/pjms.41.6.11293

**Published:** 2025-06

**Authors:** Qian-Yi Huang, Ya-Xin Huang, Wan-Yang Mu, Peng-Cheng Li

**Affiliations:** 1 Qian-Yi Huang, MD Department of Transfusion, Beijing Anzhen Nanchong Hospital, Capital Medical University & Nanchong Central Hospital, 97, South Renmin Road, Shunqing District, Nanchong, Sichuan, China; 2 Ya-Xin Huang, MD Department of Transfusion, Beijing Anzhen Nanchong Hospital, Capital Medical University & Nanchong Central Hospital, 97, South Renmin Road, Shunqing District, Nanchong, Sichuan, China; 3 Wan-Yang Mu, BA Department of Transfusion, Beijing Anzhen Nanchong Hospital, Capital Medical University & Nanchong Central Hospital, 97, South Renmin Road, Shunqing District, Nanchong, Sichuan, China; 4 Peng-Cheng Li, MD Department of Neurosurgery, Beijing Anzhen Nanchong Hospital, Capital Medical University & Nanchong Central Hospital, 97, South Renmin Road, Shunqing District, Nanchong, Sichuan, China

**Keywords:** Meta-analysis, Ovarian cancer, Perioperative blood transfusion, Prognosis

## Abstract

**Background and Objective:**

Perioperative blood transfusion (PBT) is a common procedure in ovarian cancer (OC) surgery, which may be a deleterious prognosis predictor. Therefore, this study aimed to assess the impact of PBT on survival indicators in OC patients using meta-analysis.

**Methods:**

This study was performed in adherence to the Preferred Reporting Items for Systematic Review and Meta-Analyses (PRISMA) guidelines. Relevant articles were collected by searching the PubMed, Embase, Google Scholar, and Cochrane Library databases up to September 30, 2024. A random-effects model was used to calculate pooled hazard ratios (HRs). Sensitivity analysis, sub-analysis, and publication bias were evaluated.

**Results:**

This study included nine studies, including 3,727 OC patients. 53.4% of the patients received transfusions. Overall, PBT was associated with shorter overall survival (OS), with pooled HRs of 1.44 (95% CI 1.21–1.71; P < 0.001, I^2^ = 60.4%) and 1.55 (95% CI 1.17–2.06; P < 0.001, I^2^ = 79.5%) for OS and progression-free survival (PFS), respectively. This result was also obtained using sensitivity analysis. Subgroup analysis revealed the greater impact of PBT on OS in the early OC stage than in the late stage.

**Conclusions:**

The patients receiving PBT exhibited lower survival after OC surgery, and this impact was more pronounced in early-stage patients.

## INTRODUCTION

Ovarian cancer (OC) is considered one of the most lethal gynecologic malignancies, accounting for approximately 250,000 new cases and 140,000 deaths worldwide per year.^1^,^2^ Ovarian cancer treatment consists of a combination of surgery and chemotherapy. Chishti U et al. observed that for stage III-IV OC patients, there was no significant difference in survival rate and perioperative complications between initial tumor reduction surgery and neoadjuvant chemotherapy.^3^ Postoperative anemia with a relatively high intraoperative blood loss is frequent and postoperative cytotoxic chemotherapy potentially worsens anemia. Therefore, blood transfusion in these patients is common in an attempt to correct anemia.

The surgical complexity of OC leads to a high percentage of patients in need of perioperative blood transfusion (PBT), with rates ranging from 42 to 77%. Although blood transfusions can be critical in maintaining hemodynamic stability in acute hemorrhage, some reports revealed that allogeneic blood transfusions produce immunosuppression, potentially leading to a faster tumor recurrence. PBT was associated with poor short and long-term perioperative outcomes among patients who underwent surgery for colorectal, gastric, and pancreatic cancer.^4^-^6^ However, studies evaluating the effects of PBT on OC recurrence are rare, and evidence-based guidelines regarding the role of blood transfusion in surgical patients are lacking.^7^

Since the incidence of transfusion in OC patients is high, it is important to evaluate the clinical indications for transfusion and identify potential clinical outcomes.^8^ Several articles have been published in the field of OC; however, their results are inconsistent. Based on multivariate and propensity score matched data, Pergialiotis et al. assessed the impact of PBT on the odds ratios (OR) of disease-free survival (DFS) and overall survival (OS) among the patients who underwent OC resection.^9^ However, the cohort studies included in their meta-analysis were very few and did not accurately reflect the progression-free survival (PFS) of patients. Therefore, this study used a meta-analysis approach to explore the differences in clinical characteristics, OS, and PFS between the OC patients who received allogeneic blood transfusions during the perioperative period and those who did not receive transfusions.

## METHODS

This study was performed in adherence to the “Preferred Reporting Items for Systematic Reviews and Meta-analyses” (“PRISMA” statement)” (as shown in Supplementary Materials 1 and 2).^10^

### Search strategy:

The relevant articles were collected from the PubMed, Embase, Google Scholar, and Cochrane Library databases. Using Boolean operators to connect the keywords and text words, the following MeSH terms were used to search the articles: “Ovarian Cancer” and [“Perioperative blood transfusion” or “Transfusion”] and [“Survival” or “Prognosis” or “follow-up studies” or “clinical significance”]. The final search was performed on September 30, 2024. Furthermore, the references of the selected research articles and reviews were manually evaluated to ensure that any other relevant articles were considered.

### Predefined outcomes:

OS was defined as the survival from the date of treatment to the date of death or the last follow-up. PFS was defined as the starting time of the treatment until disease progression according to the RECIST v1.1 and GCIG criteria.^11^ Perioperative transfusion was defined as the transfusion of blood products (red cell or fresh frozen plasma) 1 week before or 4 weeks after the operation.

### Inclusion criteria:

- Patients diagnosed with OC on the basis of pathological examination, and classified according to the current WHO guidelines.
- All OC patients must undergo curative OC resection and all original articles (randomized clinical trials, cohort studies, and case–control studies) should contain at least one of the following outcomes: postoperative all-cause mortality, postoperative cancer-related mortality, and five-year postoperative recurrence.
- Original articles providing the direct estimation of hazard ratios (HRs) and including 95% confidence intervals (CIs), or presenting Kaplan-Meier curves.

### Exclusion criteria:

- Duplicate studies
- Letters, comments, case reports, meeting records, and reviews
- Laboratory studies or animal studies; studies with sample size less than 30
- Studies lacking sufficient data and contact information of the authors.

### Data extraction and quality assessment:

Two reviewers (QH and YH) independently extracted the data from the selected studies, and a third author (WM) was involved in the selection in case of any discrepancies. The following data were extracted: first author, year of publication, country, sample size, median age, WHO grade of tumor, follow-up time, hemoglobin concentration before surgery, and types of blood products. The HRs for OC and the corresponding 95% CIs for the groups with transfusion (experimental group) and without transfusion (non-transfusion or control group) were extracted or obtained from the Kaplan-Meier curves. The HR and 95% CIs were preferentially extracted from the multivariate analysis when both univariate and multivariate analyses had been performed for the OS. The logarithm-transformed HR and variance were estimated based on the Kaplan-Meier curve using Engauge Digitizer version 12.1 when the HR was not reported.^12^ A quality review of data obtained from each study was performed based on case selection, comparability, and outcome reporting using the Newcastle-Ottawa Scale (NOS).^13^

### Statistical analysis:

Statistical analysis was performed using Stata Version 14.0 (Stata Corporation). OS was compared using HR and 95% CI between the transfusion and non-transfusion groups. HR greater than one indicated a poor prognosis in patients with OC curative resection and PBT. The heterogeneity of the studies was evaluated using the *I*^2^ metric, with *I*^2^ > 50% indicating a significant heterogeneity; thus, the analysis was performed using a random-effect model. *I*^2^ < 50% indicated non-significant heterogeneity; thus, the fixed-effect model was used.^14^ Subgroup analysis was performed to investigate the potential causes of heterogeneity according to the geographic area, sample size, median age, WHO grade, hemoglobin concentration at hospital admission, and data source. A *P*-value of less than 0.05 was considered statistically significant. A sensitivity analysis was performed to evaluate the influence of a single study on pooled HRs by omitting each study one by one since the patient characteristics and adjustments for confounding factors were not consistent in the studies. The sensitivity was low if no major change in the results was found, and the results were considered stable and reliable. In contrast, the sensitivity was high if the results after exclusion were significantly different compared to the original, and the stability of the results was low. The publication bias of the included studies was qualitatively tested using funnel plots and quantified using Begger’s and Egger’s tests.

## RESULTS

**Fig.1:**
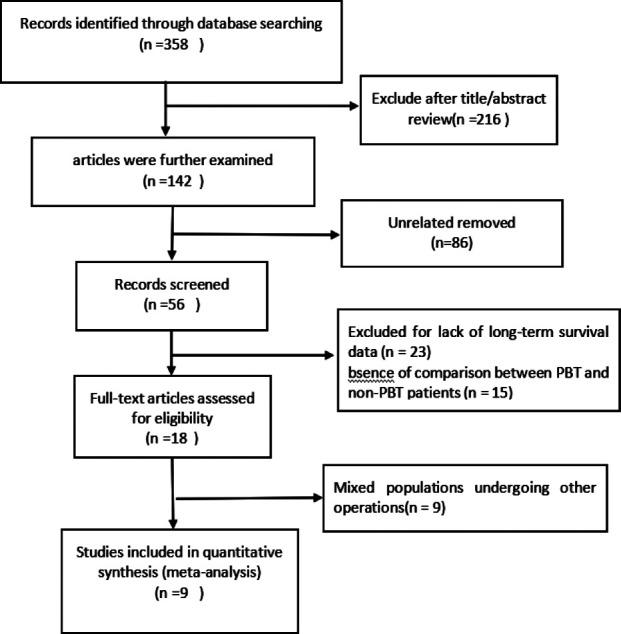
Flow diagram of search and study selection.

The literature retrieval and results are summarized in Figure 1. A total of 358 potentially relevant articles were initially selected. Among them, 216 were excluded after reading their titles and abstracts. The remaining 142 articles were further examined: 86 irrelevant studies were excluded due to the lack of long-term survival data (n = 23), the absence of comparison between PBT and non-PBT patients (n = 15), and mixed populations who were subjected to other operations (n = 9). Finally, nine studies compared the characteristics of patients who received PBT versus those who did not.^15^-^23^

### Characteristics of the studies and quality assessment:

The general characteristics of the included studies are summarized in Table-I. The selected studies were retrospective cohort studies and published from 2012 to 2023; five were from the USA^15^,^20^-^23^, two from Europe^17^,^19^, and two from Asia.^16^,^18^ These articles included 1989 PBT OC patients and 1738 non-PBT OC patients; 563 with stage I-II OC and 3158 with stage III-IV OC. The research subject of the four articles was OC patients in stages III or IV^15^,^20^,^21^,^23^, while the remaining five articles included OC patients at all stages (I-IV).^16^-^19^,^22^ Propensity score matching was used to estimate the impact of the intervention on OS in three articles.^18^-^19^,^23^ The duration of the follow-up was 20-60 months. The hemoglobin level at hospital admission was significantly lower in patients who received PBT (1.00 ± 0.20 g/d, *P* < 0.001). The HRs and 95% CIs for OS in all studies were directly extracted from 7 studies, while they were calculated from the Kaplan-Meier curves in the remaining studies. The comparative data on 5-year PFS were reported in six studies. The methodological quality in terms of population selection was good, as revealed by the NOS score of 6-9 (median 7).

**Table-I:** Baseline characteristics and survival outcomes of included studies comparing perioperative blood transfusion (PBT) and non-transfusion (Non-PBT) groups.

Author	Year	Country	Sample size (PBT/Non-PBT)	Mean age (PBT/Non-PBT)	HB(g/dl) (PBT/Non-PBT)	OS HR (95%CI)	PFS HR (95%CI)	Date source	Tumor stage (PBT/Non-PBT)		Follow-up (month)	NOS score
									I+II	III+IV		
Gildasio S	2012	America	76/60	59/59	11.2/11.4	1.37(0.91-2.09)	/	K-M curve	/	76/60	42-73	6
Lindsay L	2013	America	452/135	64.5/61.4	12.5/13.6	1.75(1.31–2.33)	1.96(1.43,2.68)	Reported	67/61	385/74	36-60	6
Joseph P	2018	America	87/44	66.4/65.6	12.0/13.0	1.62(0.88-2.99)	/	K-M curve	/	87/44	40-80	7
Oliver Hunsicker	2019	Germany	408/121	58.0/52.0	12.5/13.5	2.87(1.7-4.84)	2.71(1.94,3.77)	Reported	72/65	336/56	46-60	8
Beryl L	2019	America	136/134	/	/	1.2(0.9–1.7)	1.2(0.9–1.6)	Reported	/	136/134	/	7
Hao Zhang	2020	China	329/708	52.4/54.0	10.5/12.9	1.12(1.03–1.39)	/	Reported	67/142	262/566	24-60	7
Katharina Anic	2022	Italy	57/54	/	/	1.66 (0.95-2.93)	1.10(0.62-1.98)	Reported	7/12	46/42	/	6
Lauren S	2023	America	323/ 289	62/63	/	1.18(0.94-1.48)	1.14(0.91,1.43)	Reported	/	322/289	/	9
Yawen Zheng	2023	China	121/193	53.86/52.39	11.5/12.4	1.59(1.12-2.25)	1.63(1.22-2.18)	Reported	11/60	110/133	20-80	8

***Abbreviations:*** PBT, perioperative blood transfusion; Non-PBT, perioperative non-blood transfusion; HB, hemoglobin concentration of preoperative; OS, overall survival; PFS, progression free survival; HR, Hazard ratio; CI, Confdence interval.

### Meta-analysis of OS and PFS:

**Fig.2:**
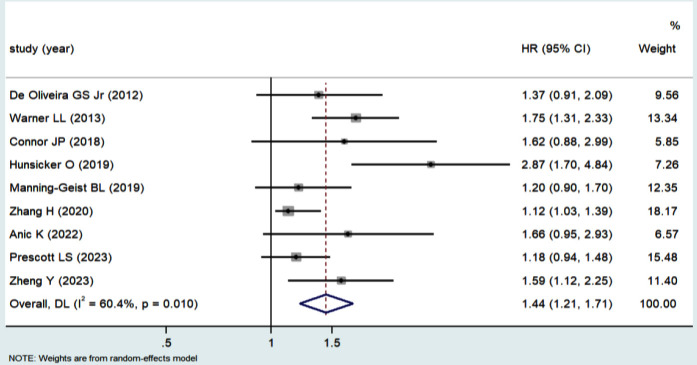
Forest plot depicting hazard ratios and 95 % confidence interval of overall survival for perioperative blood transfusion.

**Fig.3:**
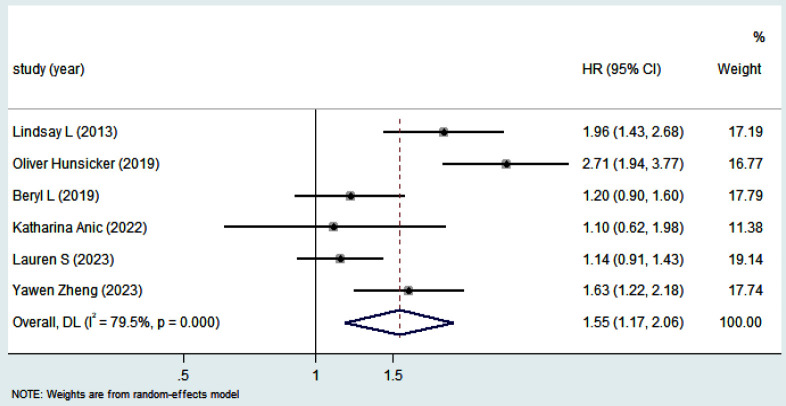
Forest plot depictinghazard ratios and 95 % confidence interval of progression free survival for perioperative blood transfusion.

**Fig.4:**
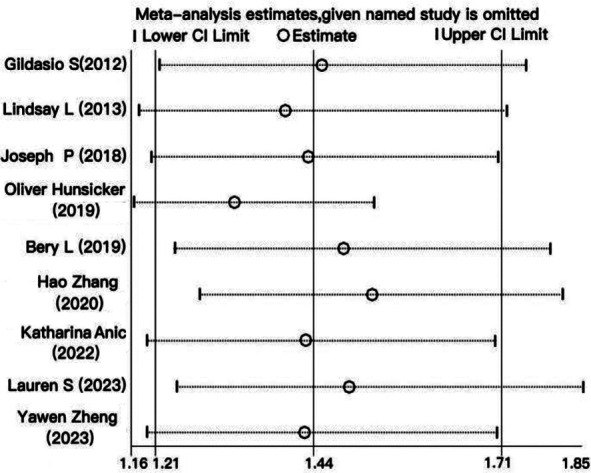
Sensitivity analysis of the relationship between Perioperative blood transfusion and overall survival.

Overall, the patients receiving PBT had a shorter OS (HR 1.44, 95% CI 1.21–1.71) (Fig.2) and PFS (HR 1.55, 95% CI 1.17–2.06) (Fig.3) compared to patients who did not receive PBT. A random model was chosen for the meta-analysis due to the significant heterogeneity among studies (Cochran’s Q, *I*^2^= 60.4%, *P* = 0.010 *vs*. Cochran’s Q, *I*^2^= 79.5%, *P* = 0.000). A sensitivity analysis was performed to assess for any potential confounding factor due to study quality, and the results showed their reliability (Fig.4).

**Fig.5:**
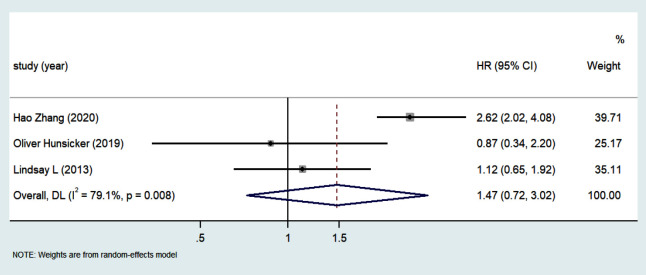
Forest plot depicting hazard ratios and 95 % confidence interval of overall survival based on propensity score matching analysis results.

The HRs of OS were summarized when the original articles provided propensity score matching analysis results. Patients who received PBT had a shorter survival rate compared with to those who did not receive PBT (HR 1.47, 95% CI 0.72-3.02) (Fig.5).

### Sub-group analysis and publication bias:

**Fig.6:**
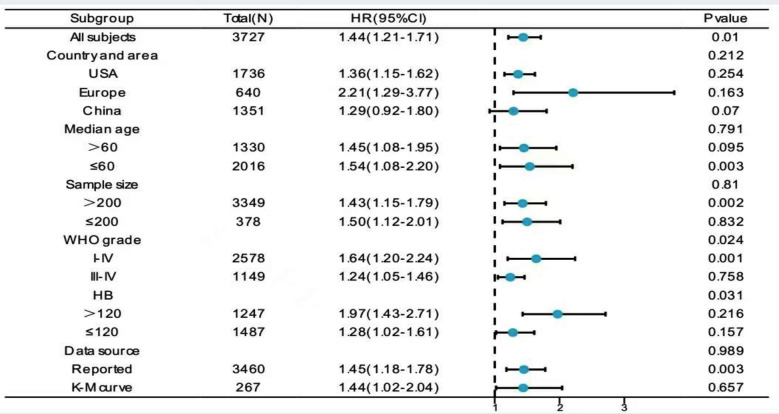
Forest plot for the association between perioperative blood transfusion and overall survival in terms of subgroup analysis.

Subgroup analysis was performed for heterogeneity values > 50% to explore its potential source, and the results are summarized in Fig.6. Patients were stratified based on the geographical area, sample size, median age, WHO grade, hemoglobin concentration before surgery, and data extraction method. The results of subgroup analysis indicate that hemoglobin concentration or WHO stages might be potential sources of heterogeneity (heterogeneity between groups: *P* < 0.05); however, the other four factors might not be related to heterogeneity. Specifically, the patients who received PBT with hemoglobin preoperative concentration > 120 g/L (HR 1.97, 95% CI 1.43, 2.71) or WHO stages I-II (HRs 1.64, 95% CI 1.20, 2.24) had a poorer prognosis. The subgroup analysis results showed a poorer prognosis of patients from the European region receiving PBT (HRs 2.21, 95% CI 1.29, 3.77), although the difference was not statistically significant.

**Fig.7:**
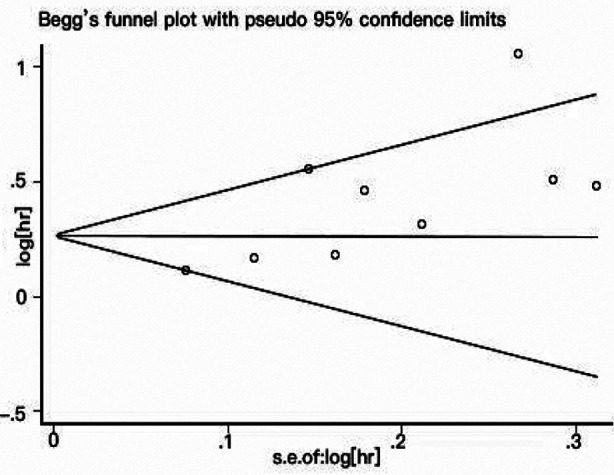
Begger’s funnel plot for the publication bias test of overall survival.

**Fig.8:**
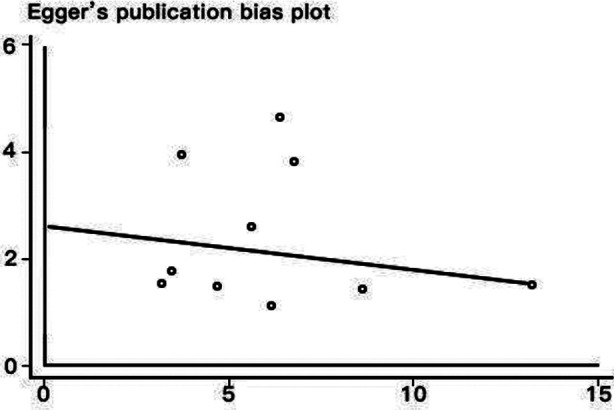
Egger’s funnel plot for the publication bias test of overall survival.

The funnel chart did not show any signs of publication bias, and Begger’s test was applied to provide statistical evidence for funnel plot symmetry. The *P*-values of Begger’s test and Egger’s test were 0.260 (Fig.7) and s 0.532 (Fig.8), respectively. Therefore, the possibility of publication bias could be ruled out in this study.

## DISCUSSION

The effects of PBT on cancer development and recurrence remain controversial. Some studies revealed that PBT has promotional effects on tumor growth and dissemination and adversely affects patients’ prognosis.^24^,^25^ In contrast, other researchers concluded the opposite, suggesting that PBT does not increase the risk of recurrence.^19^ Pergialiotis V et al.^9^ performed a meta-analysis based on five original research reporting that PBT has a significant negative impact on patients’ OS rates (OR 1.78, 95% CI 1.16-2.74) and DFS rates (OR 1.58, 95% CI 1.14-2.19). Our research is consistent with this conclusion. Strikingly, their meta-analysis on propensity score-matched populations revealed that PBT did not affect the survival outcomes of OC patients. However, their outcome should be considered with caution because of the actual reduction of the population size during the transition from the univariate to the propensity score-matched samples, which could partially obscure the results of their meta-analysis. Our meta-analysis allowed a better estimation of the effect of PBT with the combined data of multiple studies, thus minimizing the statistical error caused by the small sample size.

This study compared the outcomes between two cohorts of patients with OC: those who received PBT and those who did not receive PBT. The primary endpoints of the current study were PFS and OS. The results suggested that PBT was associated with a significantly increased risk of adverse outcomes in OC patients who underwent surgery. The HR of OS was 1.44 indicating that patients who received PBT had a 44% higher risk of poor prognosis compared to those without transfusions. The HR of PFS was 1.55, suggesting a robust link between blood transfusion and decreased survival rates or increased recurrence in this patient population. The result based on propensity score-matched populations also supported this conclusion.

The observed HR greater than one in both OS and PFS, with a confidence interval that did not cross one, underscored the negative impact of PBT on the prognosis of OC patients. Subgroup analysis for a geographic area, sample size, median age, WHO grade, and data extraction method as well as sensitivity analysis by omitting one study at a time yielded similar results. Previous studies have provided valuable insights into the mechanisms by which blood products may promote tumor recurrence or metastasis. For instance, Researchers demonstrated that transfusion of allogeneic blood products can induce immunosuppression, particularly through the downregulation of natural killer (NK) cell activity and the promotion of regulatory T cell (Treg) expansion. This immunosuppressive environment may facilitate the survival and proliferation of residual tumor cells, thereby increasing the risk of recurrence.^26^ Similarly, highlighted the role of pro-inflammatory cytokines, such as IL-6 and TNF-α, released during transfusion reactions, which can enhance tumor cell adhesion, invasion, and angiogenesis.^27^ These findings underscore the complex interplay between blood product transfusions and tumor biology, emphasizing the need for cautious clinical decision-making in cancer patients.

Additionally, several biological mechanisms explain the association between transfusions and poorer outcomes.^28^-^30^ According to Cata JP et al., surgery is a period of high vulnerability because it stimulates the release of inflammatory mediators, catecholamines, and angiogenetic activators; this coincides with a period of immunosuppression. Thus, dormant tumors or micro-metastasis (such as minimal residual disease) can grow during and after surgery and become clinically relevant metastasis. Anesthetics (such as volatile agents, dexmedetomidine, and ketamine) and analgesics (such as opioids) may also contribute to the growth of minimal residual disease or disease progression. The administration of blood products that themselves have inflammatory and immunosuppressive effects may further aggravate immunosuppression derived from the combination of surgery and anesthetic.^26^,^28^ Moreover, the inflammatory response triggered by transfused blood components may create a microenvironment conducive to tumor growth.^31^

Although the overall effect was significant, the heterogeneity among the included studies should be considered. The *I*^2^ statistic of 60.4% indicated a moderate heterogeneity, suggesting that the variability among studies might have influenced the pooled estimation. Subgroup analysis further clarified which patient groups were most affected by transfusion, indicating that OC patients with PBT who come from the European region, or WHO staging in stages I-II, or with no preoperative anemia had a poorer prognosis. First, the potential factors related to region might include differences in surgical techniques, or variations in perioperative care.

Nevertheless, it was not possible to perform a detailed analysis of these factors due to a lack of relevant data in the original article. However, our results suggested that OC patients with preoperative hemoglobin levels greater than 120 g/L had a worse prognosis, which contradicts the conclusion of the previous research. In 1999, Obermair and colleagues investigated the impact of preoperative hemoglobin levels in OC patients.^32^ Their results revealed a decreased survival in patients with OC stage I or II who were anemic (< 120 g/L) compared with those with normal hemoglobin levels. This suggests that preoperative anemia is a sign of a more aggressive or less responsive disease or that red blood cell dysfunction occurs before disease intervention and impacts the response to treatment.

Our conclusion might be influenced by the different number of patients between the high and low hemoglobin concentration subgroups, which did not match during the comparison. Xu et al. transcriptomic results analyzed by scRNA-seq delineate an ecosystemic landscape of OC at early and late stages, with a focus on the heterogeneity of its tumor microenvironment.^33^ They found that the extent of the migration of monocytes, B cells, T cells, and natural killer cells is less at the late OC stage; such factors may influence OC behavior and outcomes. Therefore, the inhibitory effect of blood transfusion on the immune function in the advanced OC stage is relatively insignificant, thus explaining why PBT has a greater impact on OC patients in the early stage. Although further research is needed to verify this result, it also suggested being cautious about performing blood transfusions in the early stages of tumors.

Based on these findings, clinicians should carefully consider the risks and benefits of PBT in OC patients. Strategies to reduce transfusion to minimize unnecessary exposure to allogeneic blood products and adverse outcomes should be considered, such as the use of iron supplements, erythropoiesis-stimulating agents, or intraoperative blood salvage, particularly in patients with early-stage disease or those at high risk of recurrence.^34^,^35^

### Limitations:

Despite the robust association found in our meta-analysis, certain limitations should be mentioned. For instance, the impact of blood transfusion may differ according to the extent of patient status, surgical resection, preoperative hemoglobin levels, adjuvant chemotherapy, or the presence of comorbidities. The characteristics of the included studies limit the ability to evaluate these factors, which were not taken into consideration. Furthermore, the heterogeneity and potential publication bias of the data might affect the validity of the results. Future research should focus on large-scale, randomized controlled trials to establish the final causal relationships and explore optimal transfusion strategies specific to OC surgery.

## CONCLUSIONS

This study demonstrates that PBT is associated with a significantly increased risk of adverse outcomes in OC surgery, especially in the OS of patients in the early OC stages. These findings underscore the importance of careful blood management and call for further investigation of personalized transfusion strategies to optimize patient prognosis.

### Authors’ Contribution:

**QH:** Conception and design, data acquisition and analysis, drafting and responsible and accountable for accuracy and integrity of the work.

**YH:** Data acquisition and analysis. Critical Review

**WM:** Literature search, Data interpretation, drafting the manuscript.

**PL:** Conception and design, data analysis and interpretation, critical revision of the manuscript.

All authors have read and approved the final version.
